# Critical Role of Zinc as Either an Antioxidant or a Prooxidant in Cellular Systems

**DOI:** 10.1155/2018/9156285

**Published:** 2018-03-20

**Authors:** Sung Ryul Lee

**Affiliations:** Department of Convergence Biomedical Science, Cardiovascular and Metabolic Disease Center, College of Medicine, Inje University, Busan, Republic of Korea

## Abstract

Zinc is recognized as an essential trace metal required for human health; its deficiency is strongly associated with neuronal and immune system defects. Although zinc is a redox-inert metal, it functions as an antioxidant through the catalytic action of copper/zinc-superoxide dismutase, stabilization of membrane structure, protection of the protein sulfhydryl groups, and upregulation of the expression of metallothionein, which possesses a metal-binding capacity and also exhibits antioxidant functions. In addition, zinc suppresses anti-inflammatory responses that would otherwise augment oxidative stress. The actions of zinc are not straightforward owing to its numerous roles in biological systems. It has been shown that zinc deficiency and zinc excess cause cellular oxidative stress. To gain insights into the dual action of zinc, as either an antioxidant or a prooxidant, and the conditions under which each role is performed, the oxidative stresses that occur in zinc deficiency and zinc overload in conjunction with the intracellular regulation of free zinc are summarized. Additionally, the regulatory role of zinc in mitochondrial homeostasis and its impact on oxidative stress are briefly addressed.

## 1. Introduction

Oxidative stress can be defined as an excessive production of reactive oxygen/nitrogen species (ROS/RNS), known as prooxidants, and/or a deficiency of enzymatic and nonenzymatic antioxidants, which are involved in the detoxification of ROS/RNS [1]. The occurrence of oxidative species is normal in a cell, but excess production occurs when the discharge of oxidants becomes too large for the cellular antioxidant defense mechanisms to detoxify or when the functioning of the antioxidant defense mechanisms is perturbed. Excessive oxidants cause alterations to the normal structure and function of DNA, lipids, and proteins, which trigger mutagenesis and oxidative damage in the cell. Therefore, excessive oxidative stress can generally be considered both a cause and an effect of numerous pathological conditions, such as cancer, neurodegeneration, cardiovascular diseases, diabetes, and kidney diseases, as discussed elsewhere [2–4]. It has been suggested that increased oxidative stress is involved in aging [5]. To combat excessive oxidative stress, various natural or synthetic antioxidants have been evaluated to prevent or attenuate the pathological transition [2, 6–8].

In living systems, various metals are involved in a wide variety of biological processes through their action as catalytic and structural components, as discussed elsewhere [7, 9]. Redox-active metals (e.g., copper and iron) participate in cycling reactions through the transfer of electrons between metals and as substrates to perform redox homeostasis in cellular biochemical reactions. Even these essential metals can cause uncontrolled oxidative stress when their regulation is disturbed [6, 10]. Toxic metals, such as arsenic and cadmium, which have no apparent biological function, can interact strongly with proteins and DNA [6, 7] and cause site-specific damage that results in the conformational change of the proteins and DNA and the excessive production of metal-mediated ROS and RNS [11]. Given the abundant anthropogenic influences in the environment, people may be exposed to these metals via the inhalation of contaminated air, the dietary intake of plant-derived food, and drinking water [12]. Industrial zinc use has increased over time; currently, it is used in galvanization, zinc-based alloys, brass, and bronze [13]. Zinc is also used for dental, medical, and household purposes [13]. Previously, the importance of zinc in health and disease has been largely studied from the perspective of severe deficiency with obvious clinical signs [14, 15]. In contrast with iron, copper, mercury, cadmium, and other metals that accumulate in tissues and produce toxic effects [16], there are fewer disorders associated with the excessive accumulation of zinc [14]. In the long-term high-dose supplementation of zinc, many of the toxic effects associated with zinc are a result of copper deficiency [17, 18]. Zinc possesses bactericidal properties at low concentrations. This is known as the oligodynamic effect and was identified by Karl Wilhelm von Nägeli through the mechanisms of oxidation-reduction reactions and the intracellular accumulation of ions in bacteria [19]. Moreover, clinical evidence has emphasized the importance of zinc in autodebridement, anti-infective action, and the promotion of epithelialization [20]. It has also been proven that topical administration of zinc in ointments or in bandages was better for disinfection and stimulation of wound healing than oral administration [20].

Zinc belongs to the divalent metals in group 12 of the periodic table and is normally colorless. Unlike other bioactive metals such as iron (ferric state (Fe^3+^) or ferrous state (Fe^2+^)) and copper (cuprous state (Cu^+^) and cupric state (Cu^2+^)), zinc is stable as a divalent cation (Zn^2+^) and does not directly undergo redox reactions owing to its filled *d* shell [21]. Compounds containing Zn^1+^ are rare and require bulky ligands to stabilize the low oxidation state. Zn^2+^ cannot donate or receive a free electron and it is therefore redox inert; it is not considered an antioxidant in the traditional sense. Instead, Zn^2+^ can function as an efficient Lewis acid and is often integrated with four ligands into a tetrahedral array with side chains of amino acids such as aspartic acid, glutamic acid, cysteine, and histidine [21, 22]. As indicated by reviews of chronological research events in zinc biology [23, 24], the reported biological roles of zinc in health and disease have rapidly increased. Adults have approximately 1.4–2.3 g of zinc in their body and its content varies significantly between tissues. In this review, Zn^2+^ refers to the reactive pool of zinc, but “zinc” refers to the total zinc content, which encompasses all forms of zinc including the protein-bound pool (54%), the exchangeable reactive pool (44.7%), and Zn^2+^ [25]. Numerous findings have linked the induction of the enzymes involved in antioxidant defenses and with antioxidant potentials to the action of free or labile forms of Zn^2+^ [22, 26]. However, it is not easily discriminable whether the identified actions are derived from zinc or Zn^2+^. The antioxidant function of zinc was suggested previously [26] and the anti-inflammatory effects of zinc were thereafter rapidly discovered [27, 28]. Paradoxically, the oxidative stress mediated by zinc deficiency and Zn^2+^ overload has been suggested to be closely linked to neurodegeneration in Alzheimer's disease [29] and neuronal cultures [30, 31]. Neuronal death that results from excess Zn^2+^ did not occur because of exogenous zinc, but because of intracellular Zn^2+^ overload, which was mobilized and redistributed in the brain [32].

**Figure 1:**
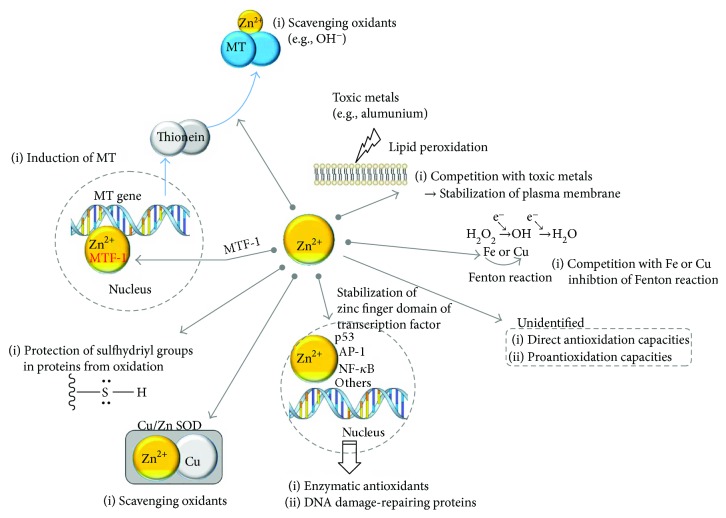
Involvement of zinc as an antioxidant. AP-1: activator protein 1; Cu: copper; Fe: iron; MT: metallothionein; MTF-1: metal-responsive transcription factor-1; NF-*κ*B: nuclear factor kappa-B; p53: tumor suppressor p53; SOD: superoxide dismutase.

**Figure 2:**
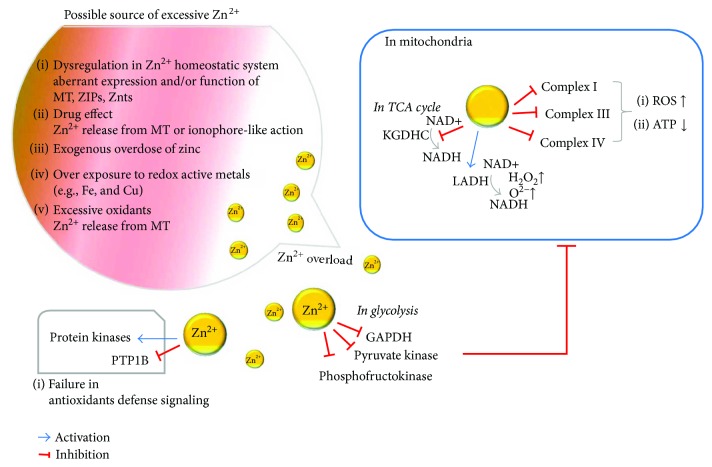
Involvement of zinc as a prooxidant. ATP: adenosine triphosphate; complex I: NADH:ubiquinone oxidoreductase; complex III: cytochrome bc1 complex; complex IV: cytochrome c oxidase; GAPDH: glyceraldehyde-3-phosphate dehydrogenase; KGDHC: alpha-ketoglutarate dehydrogenase complex; LADH: lipoamide dehydrogenase; MT: metallothionein; NADH: nicotinamide adenine dinucleotide; PTP1B: protein tyrosine phosphatase 1B; ROS: reactive oxygen species; TCA: tricarboxylic acid, (NADH); ZIP: zinc importer; ZnT: zinc transporter.

The role of zinc as either an antioxidant (Figure 1) or prooxidant (Figure 2) is not straightforward owing to the diversity and complexity of Zn^2+^ activity. The purpose of this review is to provide an insight into the dual action of zinc, which acts as either an antioxidant or a prooxidant, and the circumstances in which each function is active. First, we have briefly addressed the biochemical and nutritional aspects of zinc. Second, the underlying mechanisms of zinc homeostasis are outlined and the redox involvement of zinc is addressed in conjunction with metallothionein (MT), which is a low-molecular weight cysteine-rich protein with Zn^2+^-binding capacity [33–35]. Third, the oxidative stresses that occur in zinc deficiency and zinc overload are summarized. Fourth, the regulatory role of zinc in mitochondrial homeostasis and its impact on the oxidative stress have been discussed. Finally, we discussed the role of zinc as either an antioxidant or a prooxidant in conjunction with the intracellular regulation of free zinc.

## 2. Biochemical and Nutritional Properties of Zinc

Zn^2+^ is an essential heavy metal required by approximately 2800 macromolecules and more than 300 enzymes to build their proper structure and develop their function [36, 37]. In prokaryotes, approximately 83% of zinc proteins conduct enzymatic catalysis [38]. Eukaryotes use Zn^2+^ in diverse biological functions: zinc-related proteins in catalytic reactions (47%), DNA transcription (44%), protein transport systems (5%), and signaling pathways (3%) [38]. Zn^2+^ is also involved in stabilization of the membrane structure [39–41], and its deficiency impairs the plasma membrane functions required for platelet aggregation, osmotic protection, and various other processes [41]. Therefore, the widespread deficiency of Zn^2+^ is likely to result in major health consequences such as severe defects in growth, development, and proper functioning of the reproductive, immune, and neurosensory systems and in behavior, as discussed elsewhere [15, 24, 42]. According to the TOXNET database of the US National Library of Medicine, the oral 50% lethal dose for zinc is approximately 3 g/kg body weight, which is over 10-fold higher than that for cadmium and 50-fold higher than that for mercury [43]. There are three major routes of entry by which zinc may reach a toxic level in the human body: through the inhalation of zinc oxide as dust or fume, through the skin, or by ingestion. Several studies have demonstrated that exposure to high concentrations of zinc (up to several millimolar) or industrial exposure did not produce severe health concerns [7, 44]. Excessive oral zinc intake (over 150 mg/day) for long periods may induce copper (Cu) deficiency-like symptoms owing to the resulting inhibition of Cu uptake [45].

Zn^2+^ is hydrophilic and cannot permeate across the cytoplasmic plasma membrane and the membranes of intracellular compartments. Cellular and whole-body Zn^2+^ levels are controlled by MTs, Zn^2+^ transporters (solute carrier family 30A, ZnTs), and Zn^2+^ importers (solute carrier family 39A, ZIPs) [46, 47]. The ZnT family facilitates the mobilization of Zn^2+^ in the opposite direction of ZIP (as discussed elsewhere [48]). In addition to the ZIP family, other membrane transporter proteins, including some types of voltage-gated calcium channels, the glutamate receptor, the acetylcholine receptor, and the transient receptor potential (TRP) channel are involved in mobilization of Zn^2+^ across the cellular membrane (as discussed elsewhere [49]). In the wound healing process, extracellular Zn^2+^ released from cellular injuries can be sensed by ZnR/GPR39, which can be triggered by nanomolar concentrations of Zn^2+^, and promotes signaling that leads to epithelial repair [50]. The increased Zn^2+^ level in the cytosol will be recognized by the Zn^2+^-sensitive transcription factor, MTF-1 [51]; once Zn^2+^ has been bound, MTF-1 translocates to the nucleus to upregulate the expression of MT and ZnTs, such as ZnT1 [52, 53]. Differently, Zn^2+^ can be buffered within a “zinc muffler” via a still unidentified Zn^2+^ transporter and is then translocated into an intracellular zinc store, such as the endoplasmic reticulum (ER), Golgi apparatus, lysosomes [54, 55], or mitochondria [55, 56]. However, there is no clearly identified mechanism through which the intracellular or extracellular level of Zn^2+^ is exactly sensed.

## 3. MT and Its Involvement in the Zinc-Mediated Redox Switch

MTs have four isoforms (MT I–IV), which show tissue-specific expression. MT I and MT II are ubiquitously expressed but are transcriptionally regulated by the metal-response element-binding transcription factor-1 (MTF-1); MT III is predominantly expressed in neurons; and MT IV is dominant in the brain and epithelial tissue (as discussed elsewhere [33, 57]). In contrast to MT I/MT II, both MT III and MT IV are not inducible. MT is located in various cellular compartments, such as the nucleus, cytosol, and cellular organelles [58–60]. MT binds Zn^2+^ more tightly and at a higher concentration in comparison with other zinc proteins [34]. Four and three zinc ions are captured via their formation of *α* and *β* clusters with different affinity in MT, respectively [35, 61, 62]. It has been suggested that the specific coordination of the zinc/thiolate clusters in MT [62] will react sensitively to the redox state and thus render flexibility either in cellular availability of Zn^2+^ or the redistribution of Zn^2+^ [35]. By contrast, MT can participate in redox chemistry that does not originally result from Zn^2+^ itself, but rather from its coordination of Zn(II)-thiolate moiety [62–64]. MT is able to scavenge the hydroxyl radical (OH^−^) with 300-fold greater efficacy than glutathione [65, 66]. Moreover, the inherent activity of MT as an antioxidant may be involved in the protection against oxidative damage, such as radiation injury [66] or central nervous system pathology [67]. One of the pathways of MT degradation is its cleavage by lysosomal enzymes, such as cathepsin B, in lysosomes *in vivo* [52]. Compared with apo-MT, metal-bound MT species, such as Zn-MT and cadmium-MT, are extremely resistant to degradation. Metals protect MT against proteolysis, and the degradation of MT and metal release are likely to occur concomitantly [68].

## 4. Impact of Zn^2+^ on Mitochondrial Homeostasis and Its Involvement in Oxidative Stress

Mitochondria are the major source of ROS production [69, 70]. Therefore, it is valuable to elucidate the role of Zn^2+^ in mitochondrial functions to obtain further insight into the role of Zn^2+^ as either an antioxidant or a prooxidant. In the model of ischemia/reperfusion (I/R) injury, the effect of Zn^2+^ differs between tissues. For example, brain damage during I/R can be attenuated by Zn^2+^ scavenging [71], but cardioprotectants require Zn^2+^, as discussed elsewhere [28, 72]. Mitochondria are semiautonomous organelles capable of transcription and translation, and thus, the timely supply of Zn^2+^ must be supplied for these processes. A reduced content of Zn^2+^ can affect mitochondrial biogenesis, as matrix-localized metalloproteases that proteolytically cleave newly arrived proteins during their mitochondrial maturation require Zn^2+^ as a cofactor [73]. Zn^2+^ is a powerful inhibitor of the redox-regulated Mia40/Erv1 pathway, an essential component of the import pathway used by the small Tim proteins [74]. Thus, an aberrant level of cytosolic Zn^2+^ could suppress the correct positioning of the mitochondrial proteins required for mitochondrial biogenesis. In addition, zinc deficiency also induces ER stress response owing to accumulation of misfolded proteins that induce a vicious cycle of ER stress and oxidative stress [75, 76]. Improper supply of Zn^2+^ into mitochondria, for example, an insufficient level of ZnT6 [77] or alcoholic liver disease [78], may compromise many mitochondrial enzymes and proteins involved in mitochondrial protein processing, and thus mitochondrial ROS production can be enhanced owing to mitochondrial dysfunction.

### 4.1. Glycolysis and TCA Cycle

As shown in Figure 2, several Zn^2+^-regulated enzymes involved in glycolysis have been identified [79–81]. In addition to zinc deficiency, a high level of Zn^2+^ can inhibit glycolysis via its inhibitory effects on glyceraldehyde-3-phosphate dehydrogenase (GAPDH) [79], phosphofructokinase [80], and muscle pyruvate kinase [81]; however, these effects can be reversed by a high level of histidine. A high Zn^2+^ concentration will interrupt the Krebs cycle and mitochondrial ATP production through a decrease in the production of a reduced form of nicotinamide adenine dinucleotide (NADH) [32]. The activity of GAPDH can be inhibited indirectly, because Zn^2+^ causes a reduction in nicotinamide-adenine dinucleotide (NAD^+^) levels [82]. It should be noted that the Zn^2+^-mediated inhibition of glycolysis is prominent only in cultured neurons because of their heavy reliance on glycolysis for ATP production compared with the brain [83]. In the tricarboxylic acid (TCA) cycle, the *α*-ketoglutarate dehydrogenase complex, which is an upstream of the bc1 complex (complex III), can be suppressed in the presence of a high Zn^2+^ level [84]. Mitochondrial lipoamide dehydrogenase (LADH), which catalyzes NADH oxidation by oxygen, produces hydrogen peroxide as the major product and the superoxide radical as the minor product. As high Zn^2+^ level does not only activate LADH oxidase but also inhibits both the forward and reverse mode of LADH thiol oxidoreductase activity, abundant ROS production will occur [85].

### 4.2. Mitochondrial Respiratory Complexes

Zn^2+^ can bind slowly and progressively to the active state of complex I (NADH:ubiquinone oxidoreductase), whereas it binds rapidly and tightly to the resting state(s) [86]. At micromolar concentrations, Zn^2+^ inhibits complex I more strongly than Mg^2+^, Ca^2+^, Ba^2+^, and Mn^2+^ to Cu^2+^ or Cd^2+^ through blocking of the proton transfer to bound quinone or proton translocation. Complex II (succinate dehydrogenase (SDH)), during both the forward and reverse electron transfers, will be suppressed by exposure to low micromolar concentrations of manganese (Mn), and this suppression results in the production of mitochondrial H_2_O_2_ in rat microglial cells, but not astroglial cells [87]. However, Zn^2+^ does not suppress complex II activity. The electron bifurcation at complex III (cytochrome bc1 complex) is believed to be the most critical electron transfer steps in the respiratory chain [88]. The Zn^2+^ binding to the bc1 complex does not interfere with the binding of either of the substrates, hydroquinone or cytochrome c, but at 100–200 nM, Zn^2+^ can bind to the hydroquinone center (QP Center) of Fe-S-depleted mitochondrial bc1 complexes with high affinity and in a reversible mode; thus, Zn binding can block a protonatable group in the bc1 complex [89]. Complex IV (cytochrome c oxidase) is a membrane-bound enzyme that catalyzes the reduction of O_2_ to H_2_O and uses part of the energy released in this reaction to pump protons across the membrane. Zn^2+^ could inhibit complex IV (cytochrome c oxidase) activity in a biphasic manner [90]. There is less evidence to suggest that Zn^2+^ is directly involved in changes in complex V (the F_0_F_1_ ATP synthase complex) [91].

The inhibition of ETC by high Zn^2+^ levels may lead to cellular death either via the dissipation of the mitochondrial transmembrane potential [83, 92, 93], reduced cellular ATP levels [94], or increased ROS production [92]. The mitochondrial permeability transition pore (MPTP) is sensitive to changes in thiol redox status, and thus, Zn^2+^ can stimulate MPTP opening via interference in thiol recycling [95]. However, it is controversial as to whether Zn^2+^ can directly induce MPTP opening. Mitochondrial dysfunction is not the sole source of Zn^2+^-induced cell death, as neuronal cells can be damaged not only by compromised mitochondrial function but also by PI3K-mediated inhibition of mitochondrial movement [96]. In contrast, neuronal cell death was also mediated by H_2_O_2_-induced lysosomal membrane permeabilization owing to the intolerable accumulation of Zn^2+^ in the lysosome [97].

## 5. Role of Zinc as Either an Antioxidant or a Prooxidant in Conjunction with Regulation of Intracellular Free Zinc

Harmful effects of ROS/RNS can be prevented or slowed by nonenzymatic antioxidants (e.g., alpha-tocopherol, ascorbate, glutathione, and MT) and antioxidant enzymes (e.g., superoxide dismutase (SOD), catalase, and glutathione peroxidase) [98, 99]. The action of zinc as an antioxidant (Figure 1) is linked to zinc deficiency and disease. However, involvement of cellular zinc decreases in increased levels of ROS and that of RNS is still poorly understood. There is minimal evidence that excessive oxidation/peroxidation primarily occurs *in vivo* in tissues from zinc-deficient animals [26]. Indeed, a zinc-deficient diet will cause a reduction of food intake in rats [100, 101] and thus, multinutritional defects that result from low food intake will be included in the effects of pure zinc deficiency. Unexpectedly, dietary Zn deficiency does not impair the overall antioxidant defense capabilities in any tissue [102] and an uncompromised free radical defense system remains operational [26]. Zinc itself is not redox active, and thus, Zn^2+^ does not interact directly with ROS or with carbon-centered free radicals [26]. The possible sources of ROS in low zinc levels could be connected to (1) decreased activity of key antioxidant enzymes such as Cu/Zn-specific SOD. It is known that there is no positive correlation between Cu/Zn-SOD activity and dietary Zn intake or tissue zinc concentration. The decreased activity of SOD in zinc deficiency might not be a critical factor for oxidative stress, as the overexpression of SOD in yeast did not rescue the oxidative stress levels [103]. In conditions of nutritional stress, the change in Cu/Zn-SOD activity occurs not only through the concentration of Zn^2+^ but also through the concentration of Cu [102]. Alternatively, mismetallation in the absence of Zn^2+^ may be deleterious and yield either an inactive protein or a misfolded state prone to aggregation; (2) disturbance in the induction of MT by Zn^2+^; (3) lower protection of the free sulfhydryl groups in proteins. Zn^2+^ can protect the sulfhydryl groups in proteins against oxidation, as shown in *δ*-aminolevulinate dehydratase, dihydroorotase, the cytoskeletal protein tubulin, and Zn finger DNA-binding proteins [26]. The interaction of Zn^2+^ with sulfhydryl groups can be involved in the regulation of enzymatic activity through the prevention of intramolecular disulfide formation, which causes steric hindrance and conformational changes [26]. It is noteworthy that Zn-MT does not protect all sulfhydryl groups against oxidative stress as since Zn^2+^ will be lost from Zn^2+^-MT after reaction with OH^−^ and O_2_^−^ [26]; (4) less competition with redox-active metal ions involved in oxidative reaction. The protein structure of MT can be modified by the reaction with L-homocysteine, and homocysteinylated-MT was reported to lose function and cause increased production of ROS, chronic inflammation, and atherothrombotic disease [104]. Zn^2+^ can compete with copper or iron for certain types of binding sites, owing to the similarities in their coordination chemistry [105], and thereby suppress their ability to transfer electrons in a particular environment and cause ROS production. For example, competition of Zn^2+^ with iron and copper in the cell membrane leads to an inhibition of the NADPH oxidase enzyme, another source of O_2_^−^ and H_2_O_2_ production [106], and attenuates chronic inflammation and hyperglycemia [11, 12]; (5) dysfunction of the mitochondria and/or ER owing to an insufficiency of zinc; and (6) indirect involvement in oxidative reactions. Zn^2+^ binds selectively to NADPH, but not to NADH. Therefore, Zn^2+^ can inhibit the NADPH-mediated drug-oxidizing system [107]. Zn^2+^ can impede the formation of Fe-oxygen-enoic acid complexes, which initiates lipid peroxidation [107]. In addition, Zn^2+^ can suppress the propagation step of lipid peroxidation at phosphatidylserine, which is where aluminum and related nonredox metals (Sc^3+^, Ga^3+^, In^3+^, Be^2+^, and Y^3+^) bind preferentially [39]. Hence, Zn^2+^ performs the role of a stabilizer of macromolecules and biological membranes and minimizes their oxidative/peroxidative damage [40, 41]; although, there are some exceptions [108, 109]. The pharmacological intake of zinc can confer antioxidant-like functions, as shown in its alimentary effects against acute ethanol toxicity [110], whole-body radiation [111], isoproterenol-induced cardiac oxidative injury [112], and transition metal-mediated site-specific oxidative injury [113, 114]. It is assumed that zinc participates as an antioxidant [23], as several transcription factors [27, 115] can be upregulated by Zn^2+^ and the antioxidant, detoxifying molecules (glutathione, SOD, glutathione S-transferase, hemeoxygenase-1) and nuclear factor erythroid 2-related factor 2 (Nrf2) can be induced (Figure 1).

In addition to the antioxidant role of zinc (Figure 1), several prooxidant effects mediated by Zn^2+^ also exist and are highly associated with aberrant increases in Zn^2+^ levels (Figure 2). With the aberrant expression and function of Zn^2+^ influx and/or efflux system in conjunction with MT, intracellular Zn^2+^ overload or Zn^2+^ deficiency can occur as discussed elsewhere [48, 116–118]. In this type of Zn^2+^ overload, it will take some time until the detoxifying mechanisms in the cytosol and organelles (e.g., lysosomes and mitochondria) are compromised [119]. In the case of ischemia/reperfusion or the prevalence of oxidants such as peroxynitrite and methylisothiazolinone, the released Zn^2+^ from MT or other Zn^2+^-bound proteins render Zn^2+^ as a prooxidant as a result of protein misfolding and the subsequent accumulation of these proteins that induces oxidative stress [35, 120]. Exogenous nitric oxide (NO) or N-methyl-D-aspartate (NMDA), which increase the production of endogenous NO via receptor activation leads to peroxynitrite (ONOO^−^) formation and then Zn^2+^ release from intracellular stores, as shown in cerebrocortical neurons [121]. In cardiomyocytes, ROS and/or RNS provide major contributions to a rapid increase in intracellular Zn^2+^ levels, as a result of the mobilization of Zn^2+^ from intracellular stores. Moreover, during oxidative stress, including ultraviolet A irradiation, Zn^2+^ is released from MTs, either by nitrosylation or oxidation of the thiol ligands. Surgical injury or tissue damage, which releases Zn^2+^ from damaged cells, leads to a local increase in Zn^2+^ concentration in the neighboring cells. Certain drugs or metabolites can release Zn^2+^ from proteins, which will then cause cytotoxic effects. For example, ebselen, a selenium-containing redox drug that mimics the glutathione peroxidase, releases Zn^2+^ through the oxidation of Zn^2+^-thiolate clusters in MTs [122]. It should be mentioned that release of Zn^2+^ from Keap1 by phase II inducers, such as oxidants and electrophiles, dissociates NRF2 from keap1; thus, this mechanism can function as a sensor for the induction of antioxidant responses [123].

Excessive Zn^2+^ stimulates mitochondrial ROS production, as described in Section 4. Zn^2+^-ROS generation (produced by mitochondria, NADPH-oxidase (Nox), and other sources) can trigger further intracellular Zn^2+^ mobilization. Zn^2+^ appears to have other metabolic effects that are largely independent of the direct effects on mitochondria (Figure 2). In endothelial cells, aberrant increase in Zn^2+^ may lead to an increase in Nox1 expression, which contributes to the progression of vascular senescence [124]. Notably, senescent endothelial cells are labile to excess Zn^2+^ but are resistant to Zn^2+^ deficiency [125]. Considering the proapoptotic [126] or antiapoptotic role of Zn^2+^ [127–129], the prosenescence and proapoptotic effects of Zn^2+^ involved in vascular aging should be further elucidated in the future. In cultured neurons, Zn^2+^ has been found to trigger the activation of PKC, which results in the induction and activation of Nox1 [130] and also to induce nitric oxide synthase [131], with the resulting generation of O^2−^, NO, and peroxynitrite [131]. In contrast, Zn^2+^ itself can induce protein kinase C- (PKC-) dependent MT phosphorylation, which results in the amplification of acute Zn^2+^ increases during in vitro brain ischemia [132]. Excessive Zn^2+^ distorts the signaling pathways, and this effect may lead to disruption of cellular homeostasis (Figure 2). For example, Zn^2+^ can inhibit the protein tyrosine phosphatase 1B (PTP 1B) and consequently activate the numerous kinases targeted by PTP 1B. This uncontrolled activation of kinases results in mitochondrial dysfunction and the activation of transcription factors such as Egr-1 or Elk-1, which leads to caspase-independent cell death [133].

## 6. Conclusion

Zn^2+^ performs versatile functions involved in the structural development and catalysis of enzymes and in signal mediation in biological system. One of these functions is that the important component of Zn^2+^ in redox regulation works as an essential component in Cu/Zn SOD and antioxidant via the control of cellular signal transduction, such as gene regulation (e.g., p53, NF-*κ*B, and AP-1) and enzymatic activities. Numerous trials of Zn^2+^ supplements and topical delivery were indicated to provide beneficial impacts on health and the amelioration of pathological conditions. An adequate supply of zinc is considered essential in slowing the aging process, which in turn leads to improved cognitive functions, immune functions, stress response, and age-related neurodegenerative disorders [11]. In addition to alcoholism, zinc chelators were shown to facilitate some opioid-withdrawal signs in animals. Zinc deficiency, which affects more than 15% of the world's population, is also common among opioid consumers, and opioid-treated animals exhibit imbalances in zinc distribution [134]. As shown clinically, Zn^2+^ overload occurs in rare cases but its increase causes oxidative stress via the suppression of metabolism and mitochondrial functions. Several causes that lead to the increase of Zn^2+^ were addressed in the previous sections. Aberrant release from binding sites, such as MT, and/or from deposits in the organelles, such as mitochondria and lysosomes [55], will cause secondary oxidative stress initiated by mitochondrial and oxidant-producing enzymes. Numerous findings have suggested that excessive Zn^2+^ caused oxidative stress owing to its role as a prooxidant and eventually caused cellular death. However, it should be mentioned that numerous biomedical studies have employed irrelevant Zn^2+^ conditions, including the use of the supraphysiological concentrations of zinc, and various forms of zinc [97]. In addition, when high levels of Zn are administered orally or by injection, there may be numerous differences in the tissue distribution, metabolism, excretion, interaction, and overall homeostasis of Zn in the whole animal system [26]. In understanding of the role of Zn^2+^ as either an antioxidant or a prooxidant, the time window of Zn^2+^ effects presents another concern, as the enzymatic antioxidant systems will have a lag time for their induction whereas nonenzymatic antioxidant systems are not largely affected by Zn^2+^. In addition, an abrupt increase of Zn^2+^ will be caused either by highly oxidative conditions or by failure of Zn^2+^ homeostasis; therefore, its direct role in the antioxidant defense responses is unclear. When considering the inhibitory effect of Zn^2+^ on metabolic and mitochondrial function, augmentation of ROS production by Zn^2+^ will be more prominent. Many signaling pathways can be activated or inhibited by Zn^2+^ [24, 135, 136]. Currently, it is hard to predict where Zn^2+^ will drive a cellular response. The exclusive role of Zn^2+^ as a prooxidant cannot be separated from the innate oxidative stresses caused by other factors, as the existence of excessive oxidants exaggerates the increased Zn^2+^ in any given system. The putative effects of zinc supplementation or deficiency may be significantly affected by the concomitant changes in MT I/II expression. In addition to zinc transporters, the status of aberrant expression or mutation of MTs [48] will influence the expected antioxidant roles of Zn^2+^, because various biological functions of Zn^2+^ are cooperated with MTs, which aid in controlling the concentration of Zn^2+^ at critical sites. Indeed, the optimal amount of free Zn^2+^ and the deleterious impact of Zn^2+^ on the cellular system are complex and frequently seem to be less responsive to nutritional supplement with zinc [7]. The precise measurement of total zinc and free zinc levels in either healthy or pathological conditions allows the decision of the dose and duration of optimal zinc supplementation and maintenance. Recently, the examination of metal ionophores (e.g., dithiocarbamates and pyrithione) is in progress in cancer cell biology for their potential clinical use [137]. Metal chelators, such as desferrioxamine and tetrathiomolybdate, will suppress the availability of Zn^2+^, even at normal zinc levels. It is reasonable that more attention should be paid to the specific effects of Zn^2+^-chelator or ionophores involved in the oxidative responses in conjunction with the behavior of Zn^2+^.

In this review, we have briefly outlined why both Zn^2+^ deficiency and Zn^2+^ overload could contribute to oxidative stress. Zinc, an essential metal for life, plays important roles as an antioxidant to combat and suppress oxidative stress. In contrast, the excessive presence of Zn^2+^ triggers harmful oxidative stress. Therefore, strategies to enhance the antioxidant activity of Zn^2+^ while minimizing the induction of prooxidant effects should be the subject of future studies. Despite the numerous technical hurdles, the targeted and controlled delivery of Zn^2+^ and/or chelators to specific areas or tissues should be established for both clinical and scientific purposes.
